# Supplementary figures and images for: Methionine Sulfoxide Reductases Contribute to Anaerobic Fermentative Metabolism in Bacillus cereus
Source: Antioxidants (Basel). 2021 May 20;10(5):819. doi: 10.3390/antiox10050819 (PMC8161402; doi:10.3390/antiox10050819)

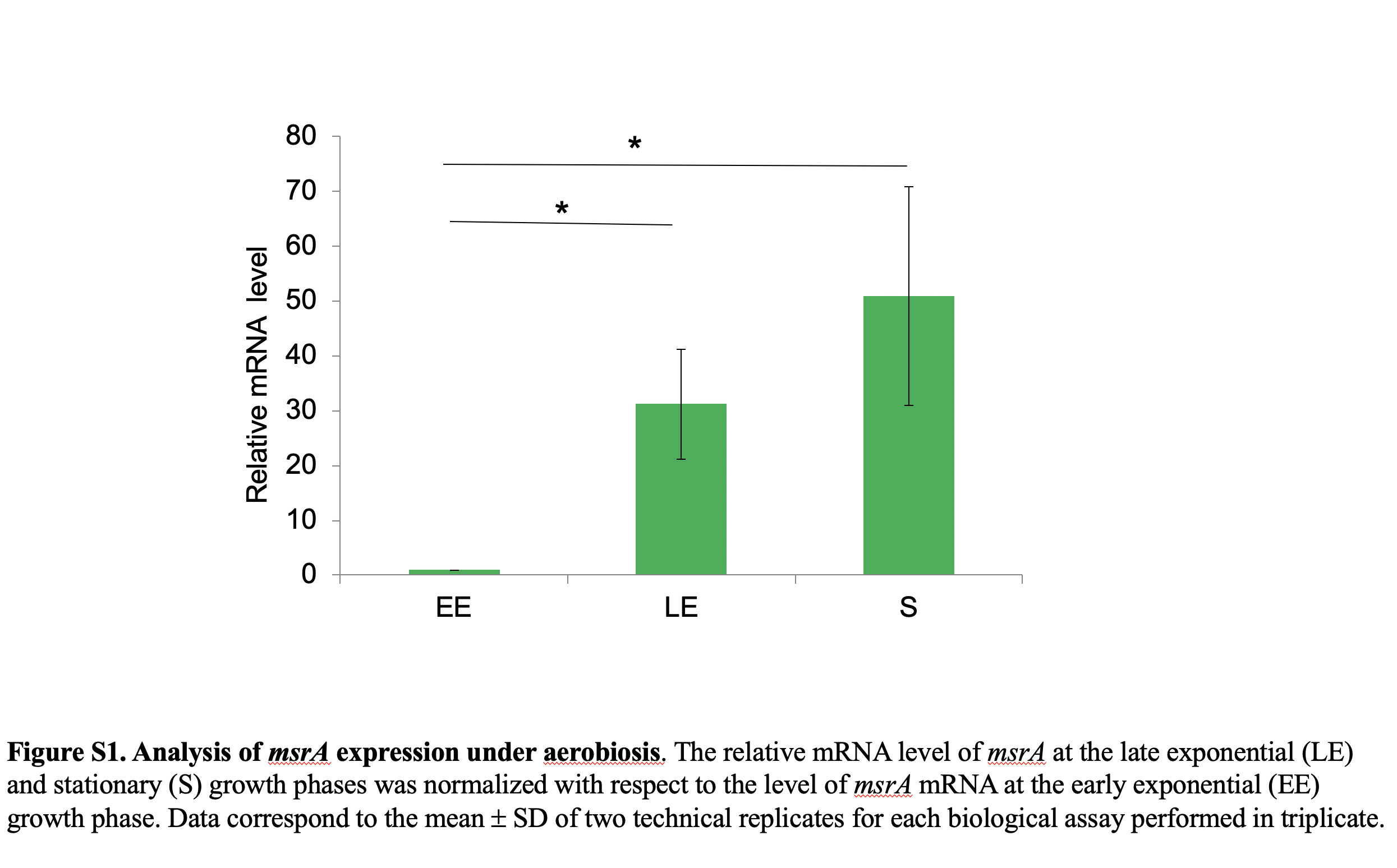

Supplement: Supplementary file 1 [file antioxidants-10-00819-s001.zip › Figure S1.tiff]
